# Relationship between weight status and anti-malarial drug efficacy and safety in children in Mali

**DOI:** 10.1186/s12936-019-2673-6

**Published:** 2019-02-18

**Authors:** Moussa Djimde, Hanen Samouda, Julien Jacobs, Hamidou Niangaly, Mamadou Tekete, Seydou B. Sombie, Erick Josephat Mgina, Bakary Fofana, Issaka Sagara, Ogobara K. Doumbo, Michel Vaillant, Abdoulaye A. Djimde

**Affiliations:** 1 Malaria Research and Training Center (MRTC), University of Sciences of Techniques and Technologies of Bamako (USTTB), Bamako, Mali; 2 Centre of Competence for Methodology and Statistics (CCMS), Luxembourg Institute of Health (LIH), Strassen, Luxembourg; 3 Population Health Department, Epidemiology and Public Health Research Unit (EPHRU), Luxembourg Institute of Health, Strassen, Luxembourg; 4 Centre National de Recherche et de Formation sur le Paludisme (CNRFP), Ouagadougou, Burkina Faso; 5 National Institute of Medical Research (NIMR), Dar es Salaam, Tanzania

**Keywords:** Malaria, Weight status, Children, *Plasmodium falciparum*, Mali

## Abstract

**Background:**

Anti-malarial treatments effectiveness remains a critical challenge for control programmes. However, when drug efficacy is established, the dose is calculated based on a predefined weight according to the patient age. Based on the hypothesis that the standard assumption of weight according to the age when administering the drug could lead to a therapeutic failure potentially due to under-dosing (in the case of overweight) or over-dosing (in case of underweight). In this study, the relationship between weight status and malaria drug efficacy in clearing current *Plasmodium falciparum* infection and preventing reinfection after treatment was investigated.

**Methods:**

Data were drown from a clinical trial conducted previously to investigate malaria drug efficacy in 749 children from Mali (2002–2004). Participants were treated either with artesunate + amodiaquine (AS + AQ, n1 = 250), artesunate + sulfadoxine–pyrimethamine (AS + SP, n2 = 248) or artesunate (AS, n3 = 251) and followed for 28 days after treatment. The World Health Organization (WHO) z-score was used to define weight status. A Chi square test was used to compare outcomes according to drugs, weight status and the dynamic of ALAT, ASAT, creatinine and haemoglobin level. Logistic regression models were developed to determine the effect of baseline parameters (weight status, aspartate transaminase, alanine aminotransferase, creatinine and haemoglobin level) on drug efficacy as per WHO criteria.

**Results:**

Without molecular correction, in AS + AQ arm, the rate of adequate clinical and parasitological response (ACPR) was higher in the group of underweight children 94.74% compared to children with normal and overweight (91.24% and 80.43% respectively, p = 0.03). After PCR correction, treatment efficacy was similar in the three groups of patients and was above 98% (p = 0.4). Overweight was observed to have no impact on recrudescence. However, it was associated with an increased risk of new infections in the (AS + AQ) arm (OR = 0.21, 95% CI [0.06; 0.86], p = 0.03).

**Conclusions:**

The findings suggest that weight deficiency has no deleterious effect on anti-malarial drug efficacy. An increase in the rate of reinfection in overweight children treated by AS + AQ should be further explored in larger studies.

## Background

Malaria is a global major public health burden with 212 million cases and 429,000 related deaths estimated in the most recent disease census [1, 2]. Based on 2015 figures, sub-Saharan Africa remains particularly affected for about 90% of the worldwide cases and deaths [3].

In Mali, malaria is the leading cause of morbidity and mortality, in particular amongst the youngest [4, 5]. In 2012, the Demographic and Health Survey reported a prevalence of 52% deaths due to malaria amongst Malian children under age of five [5], while the World Health Organization (WHO) was estimating about 17% of related child deaths [6]. One out of five Malian children die even before their 5th year [6]. Amongst the five malaria parasites species infecting humans, *Plasmodium falciparum* is the most prevalent, causing the highest morbidity and mortality [7].

The interventions currently recommended by the WHO for the management of malaria are the use of long-lasting insecticidal mosquito nets, indoor residual spraying for vector control, seasonal malaria chemoprevention (SMC), intermittent preventive treatment for malaria in infants (IPTi), a prompt access to rapid diagnostic testing (RDT) of suspected cases and the treatment of confirmed cases with effective artemisinin-based combination therapy (ACT) [8]. Artesunate + amodiaquine (AS + AQ) is one of the two artemisinin-based combinations recommended by the Malian National Malaria Control Programme and widely used in Mali and known as safe and effective [9, 10]. Artesunate + sulfadoxine–pyrimethamine (AS + SP) is one of the artemisinin-based combinations recommended by the WHO [11], although its use is discouraged. Artesunate (AS) in monotherapy has been administrated as reference treatment of uncomplicated malaria [9]. In the current study, cases enrolled were all uncomplicated falciparum malaria.

Several factors may impact clinical manifestation of malaria, such as the patient’s age [12], level of parasitaemia/virulence, particularly *P. falciparum* [13] and/or undernutrition [14]. Generally, undernutrition weakens the body system and encourages the development of infections [7], due to micronutrients deficiency and immune system impairment. It has been reported that 450,000 Malians under 5 years have suffered from moderate acute undernutrition in 2013 [15]. In the specific context of malaria infections, this may reduce malaria specific acquired immunity from childhood onwards [12, 13].

The effectiveness of anti-malarial treatments remains an ongoing challenge according to the literature [16], particularly in case of reinfection. Usually, the dose of drug to be administered is calculated based on the population weight indices if any. However, a change in weight relative to age when administering the drug could be the cause of a therapeutic failure potentially due to under-dosing (in the case of overweight) or over-dosing (in case of underweight).

The use of concomitant medications and/or the patient health conditions might also affect drugs efficacy and safety [17]. Aspartate transaminase (ASAT), alanine aminotransferase (ALAT) and creatinine might be increased, and haemoglobin (Hb) decreased before anti-malarial drug administration and could affect the treatment efficacy and safety.

Here, the efficacy and safety of two artemisinin-based combinations (AS + AQ and AS + SP) and artesunate in monotherapy were investigated according to weight status, ASAT, ALAT, creatinine and Hb level. In addition, *P. falciparum* clearance and prevention of reinfection during post-treatment follow-up in children were addressed.

## Methods

### Data handling

This work is a secondary data analysis from a previous clinical trial (http://www.pactr.org, PACTR201802003020160) conducted to investigate malaria drug efficacy in patients living in Bougoula-Hameau, Mali, over 3 years (2002–2004). Participants were randomized to receive either AS + AQ, AS + SP, or artesunate monotherapy. In total, 250 participants were treated with “AS (3 days) + AQ (3 days)”, 248 with “AS (3 days) + SP (1 day)” and 251 with “AS alone (5 days)”. The drugs were administered orally at the following dosages: AS (4 mg/kg the 1st day and 2 mg/kg other days), AQ (10 mg/kg) and SP (25 mg/kg). Patients were followed during 28 days after inclusion. Patients were seen for clinical and biological examinations every day for the first 4 days, then on days 7, 14, 21 and 28 [9].

### Weight status

Weight was measured without shoes but wearing light clothes using a Seca 761 mechanical balance graduated in kilogrammes. For infants unable to stand, the weight was measured with an adult and then the adult weight was subtracted. Height was measured in standing position using a measuring rod graduated in centimetres [18]. WHO free access software AnthroPlus [19] was used to calculate body mass index (BMI) and related z-scores, which were used as proxy of weight status. Weight status definition was based on the following cut-offs points recommended by the WHO in 2007 [20–22]:

- Underweight: BMI z-score < − 2 [0–19 years (y) old children].
- Normal weight: − 2 ≤ BMI z-score < 2 [< 5 years], − 2 ≤ BMI z-score < 1 [5–19 years].
- Overweight: 2 ≤ BMI z-score < 3 [< 5 years], 1 ≤ BMI z-score < 2 [5–19 years].
- Obesity: BMI z-score ≥ 3 [< 5 years], BMI z-score ≥ 2 [5–19 years].
- Overweight and obesity: children with overweight and children with obesity have been all aggregated in the overweight group for the analyses.

### Drug efficacy and safety

WHO 28 days in vivo protocol was used to define the efficacy of each treatment arms [23]. For molecular correction outcome, *msp1*, *msp2* and the microsatellite *ca1* were used to discriminate recrudescent parasites from reinfection [24]. Patients were classified, with and without molecular correction, having early treatment failure, late clinical failure, late parasitological failure or an adequate clinical and parasitological response [23]. Pre-treatment weight status and measures of ASAT, ALAT, creatinine and Hb level were used to predict drug efficacy. ASAT, ALAT, creatinine and Hb level were also used to monitor drug safety.

### Statistical analysis

Prevalence of participants with underweight, normal weight and overweight was analysed and descriptive statistics were calculated (percentages, median and quartiles). One way ANOVA was used to compare the anti-malarial drugs doses according to weight status. Chi square test (χ^2^) was carried out to investigate the associations between weight status, clinical and biological features at enrolment as well as treatment outcome. Logistic regression analyses were performed to analyse the effect of baseline weight status (underweight, overweight including obesity) on the efficacy of the treatment. A second logistic regression model investigated the effect of other baseline parameters, such as ALAT, ASAT, creatinine and Hb level in addition to weight status, on drug efficacy.

A Beeswarm Boxplots were used to determine median and quartiles of clinical and biological parameters according to children weight status in order to investigate the safety of the treatments. The significance, threshold was set at 0.05. The R© software version 3.5.1 (2018-07-02) was used for statistical analysis.

## Results

Baseline characteristics are described in Table 1. The distribution of treatment arms (p = 0.11) and patients gender (p = 0.95) were similar amongst weight status groups. Around 40% of under 5 years age children had an overweight, 50% had a normal weight, 10% had an underweight. Amongst older children, 71% showed a normal weight, 12% had an underweight and 17% had an overweight (p < 0.0001) (Table 1). Within the overweight group, 130 children had overweight and 114 had obesity.

**Table 1:** General characteristics at enrolment as a function of weight status

	Underweight n (%)	Normal weight n (%)	Overweight n (%)	p-value
AS + AQ arm (n = 250)	19 (7.6)	137 (54.8)	94 (37.6)	0.11
AS + SP arm (n = 248)	29 (11.69)	138 (55.65)	81 (32.66)	
AS arm (n = 251)	30 (11.95)	152 (60.56)	69 (27.49)	
Male (n = 354)	36 (10.17)	201 (56.78)	117 (33.05)	0.95
Female (n = 395)	42 (10.63)	226 (57.22)	127 (32.15)	
< 5 (n = 513)	49 (9.55)	259 (50.49)	205 (39.96)	< 0.0001
≥ 5 (n = 236)	29 (12.29)	168 (71.19)	39 (16.53)	
Temperature (°C) median (interquartile range)	38.50 (37.80, 39.18)	38.40 (37.80, 38.90)	38.65 (38.00, 39.10)	
*P. falciparum* (/µl) median (interquartile range)	17,800 (5925, 33,060)	14,850 (7162, 28,160)	17,660 (9638, 36,380)	
Haemoglobin (g/dl) median (interquartile range)	9.85 (8.48, 11.40)	10.59 (9.50, 11.80)	9.50 (8.48, 10.90)	
ASAT (UI/l) median (interquartile range)	32.20 (23.30, 46.70)	36.00 (27.70, 43.90)	38.20 (29.40, 48.30)	
ALAT (UI/l) median (interquartile range)	15.15 (11.08, 20.10)	17.00 (12.50, 23.87)	23.31 (12.00, 24.00)	
Creatinine (mg/dl) median (interquartile range)	0.52 (0.46, 0.75)	0.55 (0.48, 0.68)	0.53 (0.44, 0.66)	
Artesunate (mean ± SD in mg/kg)	58.22 ± 32.29	69.40 ± 29.39	61.57 ± 22.77	
Amodiaquine (mean ± SD in mg/kg)	165.08 ± 69.89	185.3 ± 89.99	155.82 ± 48.07	
Sulfadoxine (mean ± SD in mg/kg)	354.17 ± 185.93	444.85 ± 194.70	387.66 ± 199.75	

N: frequency; AS + AQ: artesunate plus amodiaquine; AS + SP: artesunate + sulfadoxine–pyrimethamine; AS: artesunate; Q1: 25th percentile; Q3: 75th percentile

Microscopy has shown that at day 7 only one patient in the underweight and one patient in the normal weight group had positive smear. At day 14, 5.1% of children in underweight group were carrying malaria parasite compared to 1.6% and 2.5% in the normal weight and the overweight groups, respectively (p = 0.16). At day 21, no parasite was observed in the underweight group, in contrast, blood smear was positive in 7.5% of children with normal weight and 8.4% of children with overweight (p = 0.03). At day 28 there was no significant difference in parasite carriage (p = 0.95) between the three groups.

The mean ± SD of the first doses amodiaquine administrated was lower in overweight children 155.82 ± 48.07 mg/kg compared to underweight and normal weight children with respectively 165.08 ± 69.89 and 185.3 ± 89.99 mg/kg (p = 0.04) (Table 1). The proportion of adequate clinical and parasitological response (ACPR) after treatment without molecular correction between weight categories, in AS + AQ arm, was higher in the group of children with underweight 94.74% compared to children with normal weight and overweight (91.24 and 80.43% respectively, p = 0.03). After PCR correction, treatment efficacy was similar in the three groups of body weight and was above 98% (p = 0.44). There was no difference in treatment efficacy in AS + SP and AS-alone arms according to weight status (Table 2). The medians parasite density before treatment were 17,800, 14,850 and 17,660 trophozoites/µl for children with underweight, normal weight and overweight, respectively. Baseline parasite density was higher in underweighted children compared to those with normal weight (p = 0.03).

**Table 2:** Treatment outcome without PCR correction

	Underweight (n = 22)	Normal weight (n = 137)	Overweight (n = 92)	p-value
	n (%)	n (%)	n (%)	
AS + AQ				
ACPR	18 (94.74)	125 (91.24)	74 (80.43)	0.03
ETF	0 (0)	0 (0)	1 (1.09)	–
LCF	3 (3.9)	4 (2.92)	8 (8.7)	0.08
LPF	1 (5.26)	8 (5.84)	9 (9.78)	0.49
AS + SP				
ACPR	28 (96.55)	133 (91.24)	75 (97.4)	0.92
ETF	0 (0)	0 (0)	0 (0)	–
LCF	0 (0)	2 (1.47)	1 (1.3)	0.81
LPF	1 (3.45)	1 (0.74)	1 (1.3)	0.49
AS				
ACPR	25 (83.33)	105 (69.54)	43 (63.24)	0.14
ETF	0 (0)	0 (0)	0 (0)	–
LCF	3 (10)	15 (9.93)	11 (16.17)	0.39
LPF	2 (6.67)	31 (20.53)	14 (20.59)	0.19

ACPR: adequate clinical and parasitological response; ETF: early treatment failure; LCF: late clinical failure, LPF: late parasitological failure

**Fig. 1:**
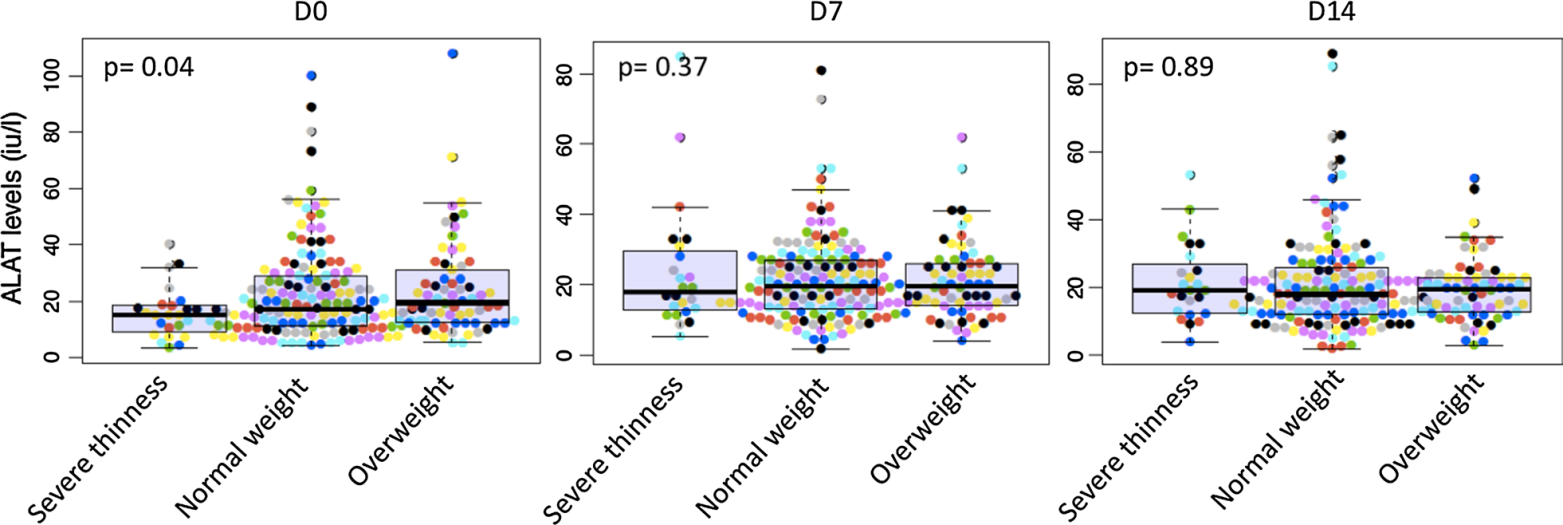
ALAT level during the follow-up in function of weight status. At day 0, one patient with normal weight and one other with overweight had respectively 740.25, and 367 U/l as ASAT level. At day 7, two patients with normal weight had very high ASAT level, respectively 484 and 237 U/l. Outlier values were not included in the figure

At enrolment the median of ALAT level (Table 1) although within normal ranges was higher in children with overweight (23.31 IU/l) compared to children with normal weight (17.00 IU/l) and 15.15 IU/l in children with underweight (p = 0.04). During the follow-up, at day 7 and day 14 the different weight status groups were similar with regards to ALAT level, p values were 0.37 and 0.89, respectively (Fig. 1).

**Fig. 2:**
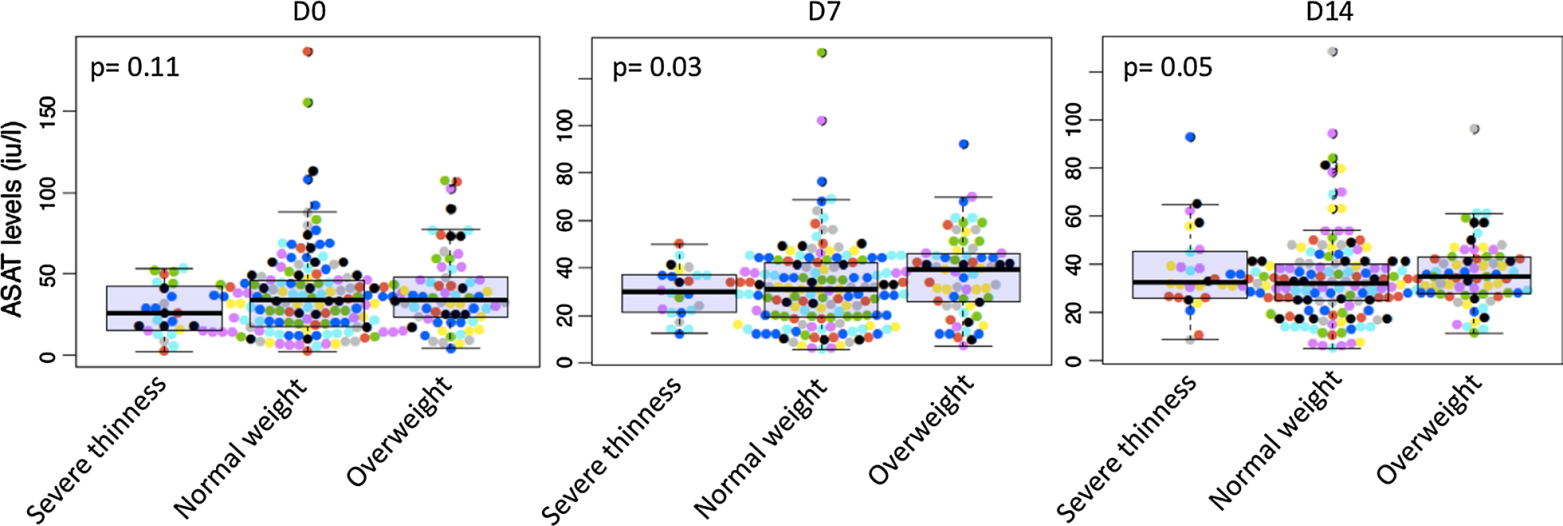
ASAT level during the follow-up in function of weight status

The ASAT levels were within normal ranges on day 7 with an highest median (Fig. 2) observed in children with overweight (37.91 IU/l) versus 30.90 IU/l in normal weight and 30.35 IU/l in underweight groups (p = 0.03). At enrolment and day 14 the three groups were similar, p = 0.11 and 0.05, respectively.

**Fig. 3:**
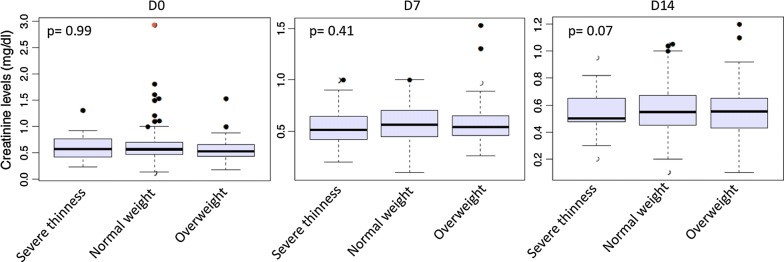
Creatinine level during the follow-up in function of weight status. At day 0, one patient with normal weight and one patient with overweight had respectively 3.84 and 4.8 mg/dl as creatinine level. At day 14 one patient with overweight had 6.7 mg/dl as creatinine level. Outlier values were not included in the figure

The level of creatinine (Fig. 3) was similar in the three groups of children (normal weight, underweight and overweight) at enrolment (p = 0.07), 7 days after the first dose of drugs (p = 0.41) and 14 days (p = 0.99).

**Fig. 4:**
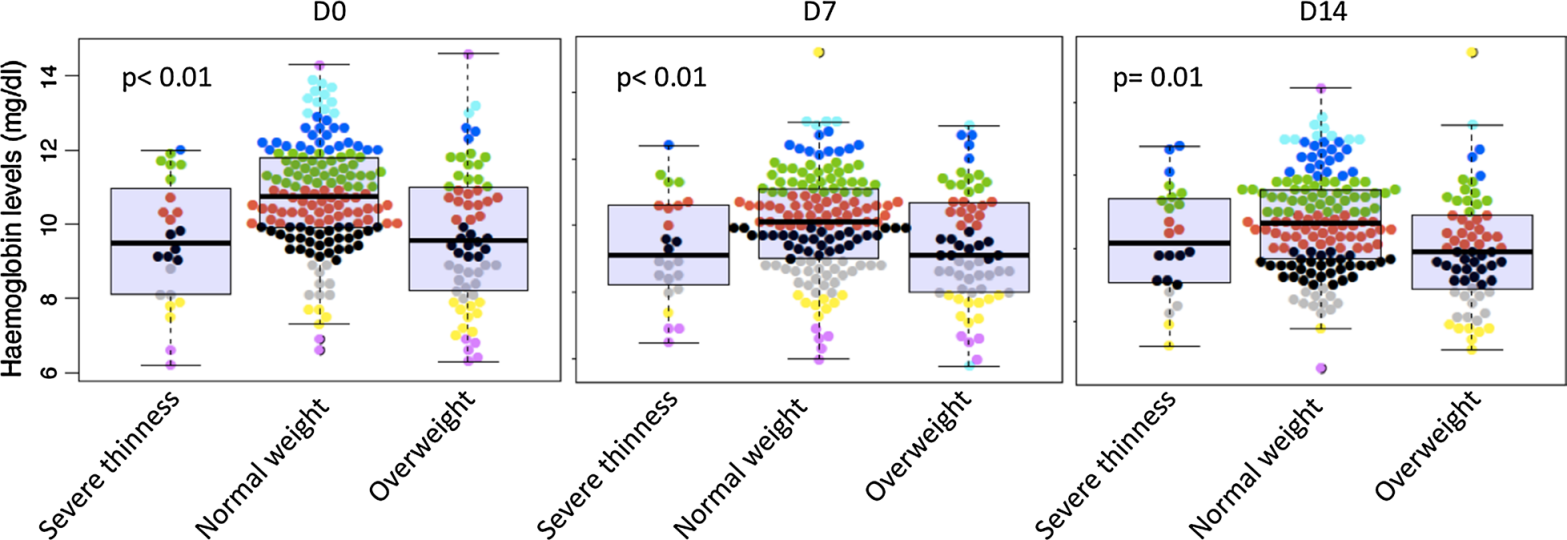
Haemoglobin level during the follow-up in function of weight status

The median (Q1, Q3) haemoglobin level at enrolment was lowest in children with overweight 9.50 g/dl (8.48, 10.90). However, the median of parasite density was similar in children according to weight status (Table 1). Throughout the follow-up, overweight and underweight children appear to have similar haemoglobin level. Children with normal weight had slightly higher haemoglobin level. The p-values were 0.001, 0.001 and 0.01 at enrolment, days 7 and 14, respectively (Fig. 4).

Logistic regression model (Table 3) showed that being overweight was associated with an increased risk of new infections in the “AS + AQ” arm (OR = 0.21, p = 0.02). In the “AS” monotherapy arm, each increase of one unit (IU/l) of ALAT level was observed to be associated with a decrease of the probability of adequate clinical and parasitological response (OR = 0.95, p = 0.02). The used model shows no association between initial weight status, baseline biological parameters and the adequate clinical and biological response after 28 days’ follow-up in the “AS + SP” treatment arm.

**Table 3:** Logistic regression model showing the effect of baseline characteristics on the PCR-uncorrected ACPR rate

	Estimate	Std. error	z value	OR (95% CI)	p-value
Artesunate + amodiaquine					
Intercept	− 0.62	2.46	− 0.25	0.54 (0.004, 83.21)	0.80
Underweight	− 0.18	1.19	− 0.15	0.84 (0.11, 17.69)	0.88
Overweight	− 1.54	0.66	− 2.34	0.21 (0.06, 0.76)	0.02
Parasitaemia	1.06e−07	1.15e−05	0.009	1 (0.99, 1)	0.99
ALAT	− 0.02	0.01	− 1.21	0.98 (0.95, 1.00)	0.22
ASAT	− 0.002	0.01	− 0.22	0.99 (0.98, 1.02)	0.82
Creatinine	0.75	1.12	0.67	2.12 (0.59, 50.83)	0.50
Hb level	0.27	0.21	1.29	1.31 (0.87, 2.01)	0.19
Artesunate + sulfadoxine–pyrimethamine					
Intercept	0.75	3.04	0.25	2.12 (0.005, 1.00e+03)	0.80
Underweight	− 0.33	1.29	− 0.26	0.72 (0.07, 16.8)	0.79
Overweight	− 0.19	1.02	0.19	1.22 (0.17, 11.2)	0.85
Parasitaemia	− 1.39e−05	1.65e−05	− 0.84	0.99 (0.99, 1)	0.40
ALAT	0.03	0.05	0.63	1.03 (0.94, 1.16)	0.53
ASAT	− 0.003	0.03	− 0.08	1 (0.95, 1.07)	0.53
Creatinine	5.91	3.22	1.84	369.65 (1.39, 4.8e+05)	0.07
Hb level	− 0.16	0.29	− 0.56	0.99 (0.47, 1.49)	0.58
Artesunate in monotherapy					
Intercept	− 3.56	1.97	− 1.80	0.03 (0.0005, 1.18)	0.07
Underweight	1.14	0.94	1.21	3.11 (0.56, 25.64)	0.23
Overweight	0.46	0.69	0.67	1.58 (0.42, 6.44)	0.50
Parasitaemia	3.73e−06	9.42e−06	0.39	1 (0.99, 1)	0.69
ALAT	− 0.046	0.02	− 2.26	0.95 (0.91, 0.99)	0.02
ASAT	0.003	0.008	1.03	1.01 (0.98, 1.02)	0.31
Creatinine	3.39	1.83	1.85	29.77 (1.71, 2088.11)	0.06
Hb level	0.24	0.17	1.49	1.27 (0.93, 1.77)	0.14

ALAT: alanine aminotransferase; ASAT: aspartate aminotransferase; Hb: haemoglobin

The multivariate analysis did not show any association between neither the baseline weight status, nor the treatment arm and the evolution of ALAT during the study (Table 4). The multivariate model (Table 4) shows higher ASAT level in children randomized into the “AS + SP” arm before treatment (p = 0.04). Putting together weight status and treatment arm, the model showed that weight deficiency is associated with high ASAT level in the “AS + SP” arm (p = 0.04). After drug administration, whatever the drug used in this study, overweight was associated with an increase of ASAT level at day 7 (p = 0.02) while no association was observed at 14 days.

**Table 4:** Multivariate analysis showing the association between baseline weight status and treatment arms and the evolution of alanine aminotransferase, aspartate aminotransferase, creatinine and haemoglobin levels

	Alanine aminotransferase (ALAT)			Aspartate aminotransferase (ASAT)			Creatinine level			Haemoglobin level		
	Estimate	Std. error	p-value	Estimate	Std. error	p-value	Estimate	Std. error	p-value	Estimate	Std. error	p-value
Enrolment: before treatment												
Intercept	17.18	3.06	2.7e−08	41.31	4.45	< 2e−16	0.63	0.09	1.44e−11	10.16	0.40	< 2e−16
Weight deficiency	3.88	3.26	0.23	− 3.62	4.75	0.44	0.05	0.09	0.59	0.52	0.43	0.23
Overweight	3.25	3.36	0.34	0.56	4.87	0.90	− 0.05	0.10	0.65	− 0.53	0.44	0.23
AS + SP	− 1.29	3.96	0.74	− 11.42	5.79	*0.04*	− 0.04	0.12	0.73	− 0.89	0.52	0.09
AS	2.31	3.93	0.56	− 3.56	5.67	0.53	− 0.07	0.13	0.58	0.05	0.52	0.93
Weight deficiency * AS + SP	1.59	4.27	0.71	12.47	6.25	*0.04*	− 0.07	0.13	0.61	0.96	0.56	0.09
Over weight * AS + SP	− 0.42	4.46	0.92	8.45	6.48	0.19	0.003	0.14	0.98	0.89	0.58	0.13
Weight deficiency * AS	− 0.24	4.24	0.32	2.63	6.11	0.66	− 0.01	0.14	0.92	− 0.34	0.56	0.54
Over weight * AS	0.72	4.48	0.87	5.04	6.46	0.43	0.01	0.15	0.92	0.11	0.59	0.86
7 Days after treatment												
Intercept	20.08	2.62	5.33e−14	31.14	3.56	< 2e−16	0.63	0.07	< 2e−16	9.62	0.37	< 2e−16
Weight deficiency	0.18	2.79	0.95	0.60	3.80	0.87	− 0.07	0.07	0.33	0.66	0.39	0.09
Overweight	1.71	2.88	0.55	8.81	3.91	*0.02*	− 0.06	0.07	0.40	− 0.38	0.40	0.35
AS + SP	− 1.89	3.39	0.58	− 0.89	4.58	0.85	− 0.07	0.08	0.36	− 0.55	0.47	0.24
AS	− 0.59	3.34	0.86	5.58	4.55	0.22	− 0.21	0.09	*0.02*	0.13	0.47	0.79
Weight deficiency * AS + SP	2.01	3.66	0.58	0.28	4.95	0.95	0.11	0.09	0.21	0.35	0.51	0.49
Overweight * AS + SP	− 0.81	3.82	0.83	− 3.14	5.16	0.54	0.07	0.09	0.46	0.54	0.53	0.31
Weight deficiency * AS	− 1.32	3.61	0.71	− 3.13	4.91	0.52	0.19	0.09	*0.05*	− 0.63	0.51	0.21
Over weight * AS	− 1.15	3.81	0.76	− 8.04	5.18	0.12	0.23	0.10	*0.03*	− 0.10	0.53	0.85
14 Days after treatment												
Intercept	22.24	2.61	< 2e−16	38.19	3.44	< 2e−16	0.58	0.07	< 2e−16	10.63	0.34	< 2e−16
Weight deficiency	− 1.07	2.79	0.70	− 4.40	3.66	0.23	− 0.02	0.07	0.75	0.26	0.37	0.48
Overweight	0.24	2.87	0.93	0.81	3.77	0.83	− 0.04	0.08	0.61	− 0.60	0.38	0.11
AS + SP	− 5.50	3.39	0.11	− 6.50	4.41	0.14	− 0.06	0.08	0.44	− 1.13	0.44	*0.01*
AS	− 3.35	3.34	0.32	− 0.94	4.35	0.83	− 0.13	0.08	0.12	− 0.58	0.44	0.19
Weight deficiency * AS + SP	3.56	3.66	0.33	6.24	4.75	0.19	0.05	0.09	0.57	1.02	0.48	*0.03*
Over weight * AS + SP	0.24	3.82	0.95	4.54	4.96	0.36	0.09	0.09	0.33	1.02	0.50	*0.04*
Weight deficiency * AS	1.55	3.61	0.67	2.07	4.68	0.66	0.10	0.09	0.26	0.12	0.47	0.81
Over weight * AS	− 1.26	3.81	0.74	− 2.43	4.94	0.62	0.15	0.09	0.13	0.55	0.50	0.27

AS + SP: artesunate + sulfadoxine–pyrimethamine; AS: artesunate used in monotherapy

Significant* p*-values are given in italic

The multivariate model (Table 4) showed an increase in creatinine level is observed at days 7 after the first dose of “AS” uptake (p = 0.02). Putting together weight status and treatment arm, the model showed an association between overweight, “AS” and the increase of creatinine level (p = 0.03). Before treatment and 14 days after drug administration no association was observed. The same model showed that at day 14 the decrease of haemoglobin level is associated with the treatment arm “AS + SP” (p = 0.01). Putting together weight status and treatment arm in the model, we observe that weight deficiency and “AS + SP” (p = 0.03); and overweight and “AS + SP” (p = 0.04) are associated with the decrease of haemoglobin level.

## Discussion

The results showed that despite the higher parasite density and lowest haemoglobin level observed in underweight children at enrolment, no association was found between underweight and decreased efficacy or safety of anti-malarial drugs used in this study. Moreover, this analysis showed that overweight tends to increase the risk of new infections in the “AS + AQ” arm. Further investigations are required to scrutinize whether the current treatment regimen of “AS + AQ” is appropriate for all children.

Out of 749 children enrolled in this study, 244 were in the overweight group and 78 children in the underweight group (Table 1). This trend might be influenced by the severe malnutrition exclusion criterion to the original clinical trial. A study conducted in the same setting in 1995 found a prevalence of chronic malnutrition in children around 25% [25].

At enrolment, malaria parasite density was higher in underweighted children. The high susceptibility to infection in malnourished children might be explained mainly by the suppression of their immunity because of undernutrition affecting the humoral and cell mediated immunity, the bactericidal activity of phagocytes and complement formation [26]. Although a higher proportion of adequate clinical and parasitological response was observed in children with weight deficiency before PCR correction in the “AS + AQ” arm, all the three groups were similar after PCR correction.

Doses of “AS + AQ” tablets were determined and administered to the children per age categories by assuming their weight according to the age. The decrease of the efficacy of treatment in children with overweight may be related to the underdosing. In contrast, the increase of treatment efficiency in the prevention of new infections in children with underweight could be explained by an effect of overdosing. Taylor et al. [27] described in a previous publication that younger and underweight children have great risk of overdosing and vice versa. In patients with overweight and obesity, iron deficiency are frequently found [28]. Studies show that the frequency of malaria was increased in patients with iron supplementation [29, 30].

The significant association observed between overweight and the increase of ASAT and creatinine levels at day 7 can be explained by the possible accumulation of drugs in fat stores. The lowest haemoglobin level (Fig. 4) was observed in children with weight deficiency before treatment. Ehrhardt et al. [14] reported that anaemia (Hb level < 11 g/dl) was associated with young age, parasite density and malnutrition. Müller et al. [31] showed that anaemia was significantly associated with malnutrition. The current analysis found that the uptake of “AS + SP” is associated with a decrease of haemoglobin level more likely du to the role of sulfadoxine and pyrimethamine inhibiting the dihydropteroate synthase and dihydrofolate reductase respectively, both involved in haemoglobin biosynthesis. In combination, their synergy is very high [32].

## Conclusion

Weight deficiency has no deleterious effect on the efficacy of anti-malarial drugs. Underweight status did not affect the drugs tolerability. The decrease of the efficacy of treatment in overweight children treated with artesunate + amodiaquine could be related to an under dosing and should be further explored in larger studies.

## Abbreviations

ACPR: adequate clinical and parasitological response
ACT: artemisinin-based combination therapy
ALAT: alanine aminotransferase
AQ: amodiaquine
AS: artesunate
ASAT: aspartate transaminase
BMI: body mass index
ca1: carbonic anhydrase 1
CCMS: Centre of Competence for Methodology and Statistics
CNRFP: Centre National de Recherche et de Formation sur le Paludisme
EPHRU: Epidemiology and Public Health Research Unit
g/dl: grammes per decilitre
Hb: haemoglobin
IPTi: intermittent preventive treatment for malaria in infants
IU/L: international unit per litre
mg/kg: milligramme per kilogramme
MRTC: Malaria Research and Training Center
*msp1*: merozoite surface protein 1
*msp2*: merozoite surface protein 2
NIMR: National Institute of Medical Research
OR: odds ratio
PCR: polymerase chain reaction
LIH: Luxembourg Institute of Health
USTTB: University of Sciences of Techniques and Technologies of Bamako
RDT: rapid diagnostic test
SD: standard deviation
SMC: seasonal malaria chemoprevention
SP: sulfadoxine–Pyrimethamine
WHO: World Health Organization
χ^2^: Chi square test
